# Preclinical stress originates in the rat optic nerve head during development of autoimmune optic neuritis

**DOI:** 10.1002/glia.23560

**Published:** 2018-12-21

**Authors:** Aleksandar Stojic, Jovana Bojcevski, Sarah K. Williams, Carlos Bas‐Orth, Stefan Nessler, Christopher Linington, Ricarda Diem, Richard Fairless

**Affiliations:** ^1^ Department of Neurology University Clinic Heidelberg Heidelberg Germany; ^2^ Institute of Anatomy and Cell Biology, University of Heidelberg Heidelberg Germany; ^3^ Institute for Neuropathology, University Medical Center Göttingen Göttingen Germany; ^4^ Institute of Infection, Immunity and Inflammation, University of Glasgow Glasgow UK

**Keywords:** auto‐antibody, EAE, oligodendrocyte, optic nerve head, optic neuritis, αB‐crystallin

## Abstract

Optic neuritis is a common manifestation of multiple sclerosis, an inflammatory demyelinating disease of the CNS. Although it is the presenting symptom in many cases, the initial events are currently unknown. However, in the earliest stages of autoimmune optic neuritis in rats, pathological changes are already apparent such as microglial activation and disturbances in myelin ultrastructure of the optic nerves. αB‐crystallin is a heat‐shock protein induced in cells undergoing cellular stress and has been reported to be up‐regulated in both multiple sclerosis and its animal model, experimental autoimmune encephalomyelitis. Therefore, we wished to investigate the timing and localization of its expression in autoimmune optic neuritis. Although loss of oligodendrocytes was not observed until the later disease stages accompanying immune cell infiltration and demyelination, an increase in oligodendrocyte αB‐crystallin was observed during the preclinical stages. This was most pronounced within the optic nerve head and was associated with areas of IgG deposition. Since treatment of isolated oligodendrocytes with sera from myelin oligodendrocyte glycoprotein (MOG)‐immunized animals induced an increase in αB‐crystallin expression, as did passive transfer of sera from MOG‐immunized animals to unimmunized recipients, we propose that the partially permeable blood–brain barrier of the optic nerve head may present an opportunity for blood‐borne components such as anti‐MOG antibodies to come into contact with oligodendrocytes as one of the earliest events in disease development.

## 1 INTRODUCTION

Optic neuritis is a common first presenting symptom of the autoimmune condition of multiple sclerosis (MS) occurring in about 25% of cases (Toosy, Mason, & Miller, 2014); and following an initial diagnosis of clinically isolated syndrome, it can be a major prognostic for subsequent progression to clinically definite MS (Miller, Barkhof, Montalban, Thompson, & Filippi, 2005). Optic neuritis, defined as inflammation of the optic nerves leading to visual impairment, is characterized by immune cell infiltration and subsequent demyelination. It can be modeled in rodents such as Brown Norway (BN) rats that are immunized with myelin oligodendrocyte glycoprotein (MOG) (Meyer et al., 2001; Stefferl et al., 1999; Storch et al., 1998). These animals, after a delay of around 12 days in which the immune response evolves, develop spinal cord symptoms typical of experimental autoimmune encephalomyelitis (EAE), with concurrent optic nerve inflammation. BN rats also have a strong anti‐MOG antibody response (Stefferl et al., 1999), which may also demonstrate the relevance of this model for other neurological diseases such as pediatric MS, subgroups of neuromyelitis optica and cases of anti‐NMDA‐receptor encephalitis where such antibodies are present (Havla et al., 2017).

We have previously reported that one of the key parameters of BN rat autoimmune optic neuritis (AON), namely degeneration of retinal ganglion cells whose axons comprise the optic nerve, is not only prevalent but also precedes immune cell infiltration of the optic nerves by several days (Fairless et al., 2012; Hobom et al., 2004). However, the early disease processes occurring during this preclinical stage are currently not fully understood, although increased numbers of activated microglia residing in both the retina and optic nerves have been detected.

To investigate the preclinical disease pathology in more detail, we have investigated the expression of αB‐crystallin, a member of the small heat shock family of proteins, as a marker of cellular stress. Previous studies of autoimmune demyelinating diseases have reported that its expression is robustly up‐regulated both in MS (Bajramovic et al., 2000; Peferoen et al., 2015) and EAE (Chabas et al., 2001; Ousman et al., 2007). In particular, αB‐crystallin has been reported to be expressed early during MS pathology, being observed within both developing lesions (Bajramovic, Lassmann, & van Noort, 1997) and in the normal‐appearing white matter (Van Noort et al., 2010), where it may represent an early stress response. Due to the similarities of the preactive lesion with the pathology of optic nerves during the preclinical stage of AON, namely microglial activation in the absence of demyelination and leukocyte infiltration, we wished to determine whether αB‐crystallin is similarly expressed in the optic nerves of MOG‐immunized BN rats, and whether, as an early marker of stress, it can help reveal the anatomy of vulnerability within the optic system.

## 2 MATERIALS AND METHODS

### 2.1 Animals

Female Brown Norway (BN, RRID:RGD_737972) rats (8–10 weeks old), used for MOG‐EAE experiments, and Sprague Dawley (SD, RRID:RGD_734476) rat pups (postnatal day two; P2), for cultures of primary glial cells, were obtained from Charles River (Sulzfeld, Germany) and kept under environmentally controlled conditions in the absence of pathogens with free access to food and water. All experiments that involved animal use were performed in compliance with the relevant laws and institutional guidelines of Baden–Württemberg.

### 2.2 Induction and evaluation of MOG‐EAE

BN rats were immunized with whole recombinant rat MOG (a kind gift of Prof. Christine Stadelmann, Department of Neuropathology, University of Göttingen). Rats were injected intradermally at the base of the tail with an emulsion (200 μl) containing 100 μg MOG in saline mixed 1:1 with complete Freund's adjuvant (CFA; Sigma‐Aldrich, St. Louis, MO) containing 200 μg of heat‐inactivated *Mycobacterium tuberculosis* H37RA (Difco Microbiology, Lawrence, KS). Sham‐immunized rats received the same volume of emulsion but without MOG, and together with healthy animals served as controls. Unless indicated otherwise, sham animals were taken at 14 days post immunization. Rats were scored daily for clinical signs of EAE as previously described (Meyer et al., 2001).

### 2.3 Passive sera transfer and depletion of anti‐MOG antibodies

Blood was collected from donor rats (MOG or sham‐immunized) by heart puncture and stored overnight at 4 °C to coagulate. Sera were then isolated by centrifugation (15,000*g* for 15 min at 4 °C) and a sample was taken for MOG‐ELISA analysis, before concentration by further centrifugation using Amicon® Ultra 15 ml centrifugal filters (3 kDa membrane; Millipore, Darmstadt, Germany) at 3000*g* at 4 °C to achieve a volume of approximately 1,000 μl per donor rat. Concentrated sera was then injected intravenously via the tail vein of naïve recipient BN rats under isofluorane anesthesia at day 0 and then a repeat injection was given on day 3 post‐serum‐transfer (pst). Blood samples were collected from recipient animals prior to and at days 1, 3, and 5 pst. At day 5 pst, blood was collected by heart puncture, and animals were perfused with 4% paraformaldehyde (PFA).

To deplete the sera of anti‐MOG antibodies, cyanogen bromide‐activated Sepharose 4B (GE Healthcare, Chicago, IL) was used according to the manufacturer's instructions. Briefly, Sepharose 4B was coupled to rat recombinant MOG protein by overnight incubation at 4 °C in coupling buffer (0.2 M NaHCO_3_, 0.5 M NaCl, pH 8.3) with subsequent washing and blocking of unreacted binding sites. MOG‐coupled Sepharose was then recovered by centrifugation at 3000*g* for 5 min before incubation with sera (using the following ratios: 268 mg Sepharose 4B coupled to 1.5 mg MOG per 1.6 ml of sera) for 1 hr at 4 °C. The resin was subsequently removed from sera by centrifugation at 3000*g* for 5 min. Successful depletion of anti‐MOG antibodies from EAE sera was confirmed by both ELISA and immunostaining of mature oligodendrocyte cultures.

### 2.4 Cell culture of primary oligodendrocytes

Primary oligodendrocytes were isolated from cortices of P2 SD pups using commercial anti‐O4‐coated magnetic beads and separation columns (Miltenyi Biotec, Bergisch Gladbach, Germany). Briefly, brains were dissected and meninges were removed before isolation of cortices which were mechanically disrupted and dissociated using 5% trypsin (Sigma‐Aldrich) and 500 U DNase 1 (Roche, Mannheim, Germany) before purification according to the manufacturer's instructions. The O4‐positive cell fraction was then seeded on either poly‐d‐lysine (PDL, Sigma‐Aldrich)‐coated 6‐well plates (100,000 cells/well for lysates) or 24‐well plates (10,000 cells/well for immunocytochemistry). Cells were grown for 7 days in proliferation medium before switching to differentiation medium for an additional week. Proliferation media consisted of a basic media (RPMI 1640 medium supplemented with 1% Sato's medium, 1% bovine pancreas insulin (0.5 mg/ml; Sigma), 1% Na‐pyruvate (100 mM; Sigma‐Aldrich), 2% l‐glutamine (2 mM; Sigma‐Aldrich) and 1% Pen‐Strep (10,000 U/ml; Gibco), supplemented with 0.1% platelet‐derived growth factor (PDGF, rat recombinant, 10 ng/ml; R&D systems, Minneapolis, MN) and 0.15% basic fibroblast growth factor (bFGF, human recombinant, 10 ng/ml; PeproTech Inc., Rocky Hill, NJ). Differentiation medium consisted of basic medium supplemented with 0.1% ciliary neurotrophic factor (CNTF, rat recombinant, 10 ng/ml; PeproTech Inc.), 0.1% N‐acetyl‐l‐cysteine (3 mg/ml; Sigma) and 1% triiodothyronine (T3, 15 nM; Sigma‐Aldrich). Purity of resultant cultures was assessed by Olig2 staining (with average purity greater than 75%), and differentiation status was identified both morphologically and using antibodies against MOG and CNPase.

Treatment of oligodendrocytes to induce stress was performed with either 100 μM H_2_O_2_, 100 μM Sin‐1 or isolated sera diluted as indicated. Following 90 min exposure, cells were washed and left to recover overnight in fresh media before fixation and immunostaining.

To assess cell survival and the generation of reactive oxygen species, commercial kits (LIVE/DEAD Fixable Dead cell stain kit and CellROX Green Reagent, respectively, both from Thermo Fischer Scientific, Waltham, MA) were used according to the manufacturer's instructions.

### 2.5 Optic nerve histopathology

Following perfusion of rats with 4% PFA, optic nerves with attached eyes were carefully dissected to maintain an intact optic nerve head. Tissue was postfixed overnight, cryoprotected in 30% sucrose (Applichem GmbH, Darmstadt, Germany) in PBS overnight, and then frozen in Cryoblock embedding medium (Medite, Burgdorf, Germany) using isopentane (Acros Organics BVBA, Geel, Belgium) cooled with liquid nitrogen. Eight micrometers thick longitudinal sections were cut using a cryostat (Leica, Wetzlar, Germany) at −20 °C, and sections transferred to SuperFrost® Plus microscope slides (Thermo Fischer Scientific,) before storage at −20 °C. Demyelination of optic nerves was assessed using Luxol‐fast blue (LFB) staining with hematoxylin counter‐staining, and immunohistochemistry was performed as described below.

### 2.6 Immunolabeling

For immunohistochemistry, antigen retrieval was performed on optic nerve sections where necessary (Olig2, MBP, and αB‐crystallin) by incubation in heated (~80 °C) 0.2% citrate buffer (pH 6.0) for 15 min, before being left to cool. For immunocytochemistry, cells were permeablized with either 0.1% Tween 20 in PBS for detection of cellular antigens, or with 0.1% Triton X‐100 in PBS for nuclear antigens (i.e., Olig2). Blocking was then performed using 10% normal goat serum before overnight incubation with primary antibodies at 4 °C. The following primary antibodies were used: olig2 (1:500; Millipore, Cat# AB9610, RRID: AB_570666); αB‐crystallin (1:1000; clone 1B6.1‐3G4; Abcam, Cambridge, UK, Cat# ab13496, RRID: AB_300400); GFAP (1:500; Sigma‐Aldrich, Cat# G9269, RRID: AB_477035); Iba‐1 (1:1000; Wako Chemicals, Neuss, Germany, Cat# 019–19,741, RRID: AB_839504); MBP (1:300; Sigma‐Aldrich, Cat# M3821, RRID: AB_1841021); MOG (1:500; clone 8‐18C5; Millipore, Cat# MAB5680, RRID: AB_1587278) and CNPase (1:200, BioLegend, San Diego, CA, Cat# 836404; RRID: AB_2566639). Appropriate secondary antibodies were then used (anti‐mouse Cy3, 1:400, Jackson ImmunoResearch Laboratories, West Grove, PA, Cat# 115–165‐166, RRID: AB_143165; anti‐rabbit A488, 1:400, Thermo Fischer Scientific, Cat# A‐11008, RRID: AB_143165) before mounting in anti‐fade Vectashield Mounting Medium (Vector Laboratories, Burlingame, CA) containing 4′,6‐diamidino‐2‐phenylindole (DAPI).

To immunolabel oligodendrocyte cultures with harvested sera, sera were added to the culture media at 1% and incubated for 30 min at 37 °C before washing and fixation. Subsequently cells underwent the same staining procedure as outlined above.

IgG antibody deposition in the optic nerves was visualized by incubation in a biotinylated goat anti‐rat secondary antibody (1:200, Vector Laboratories, Cat# BA‐4001, RRID: AB_10015300), followed by streptavidin‐Alexafluor 555 (1:700, Thermo Fischer Scientific, Cat# S32355, RRID: AB_2571525). For terminal dUTP nick end labeling (TUNEL) staining, a protocol was used as previously reported (Stojic et al., 2017), with subsequent co‐staining with antibodies being applied afterward.

Fluorescent microscopy and image acquisition were performed using either a conventional Nikon Eclipse 80i microscope (Nikon GmbH, Düsseldorf, Germany) or a LSM 700 confocal microscope (Zeiss, Oberkochen, Germany).

### 2.7 Western blotting

Optic nerve lysates were prepared by mechanical homogenization with ice‐cold lysis buffer (50 mM Tris HCl, 150 mM NaCl and 1% Triton X‐100) containing Complete Protease Inhibitor Cocktail (Roche), and sonicated for 5 s before clarification by centrifugation. About 50 μg of total protein were loaded onto a 4–20% gradient Mini‐PROTEAN® TGX Stain‐Free™ Precast gels (BioRad, Hercules, CA) and separated by SDS‐PAGE.

Primary oligodendrocyte lysates were prepared from cultures following 90 min treatment with experimental serum diluted in culture media as indicated, followed by washing with PBS and 16 hr recovery in culture media before addition of ice‐cold lysis buffer. Cells were scraped off and sonicated for 10 s before clarification. About 30 μg of total protein were loaded onto a 3–15% gradient Mini‐PROTEAN® TGX Stain‐Free™ Precast gels (BioRad) and separated by SDS‐PAGE.

Proteins were subsequently transferred to a polyvinylidene difluoride membrane for labeling with appropriate antibodies. For αB‐crystallin detection, blocking was performed in 5% BSA and 0.1% Tween 20 in TBS, and incubated in anti‐αB‐crystallin primary antibody (1:1000; clone 1B6.1‐3G4; Abcam, Cat# ab13496, RRID: AB_300400), whereas for glyceraldehyde 3‐phosphate dehydrogenase (GAPDH) detection, blocking was performed in 5% milk powder and 0.1% Tween 20 in TBS, and incubated in anti‐GAPDH antibody (1:2000; clone 6C5, Millipore, Cat# MAB374, RRID: AB_2107445). Visualization was performed using sheep anti‐mouse HRP‐conjugated secondary antibody (1:5000; GE Healthcare, Cat# NA931, RRID: AB_772210), followed by ECL Prime reagent (Amersham, Bucks, UK) and imaged using a ChemiDoc XRS+ Imaging System (BioRad).

### 2.8 MOG ELISA

To measure titers of anti‐MOG antibodies in sera samples, an enzyme‐linked immunosorbent assay (ELISA) was performed. A 96‐well PVC plate was coated by overnight incubation (4 °C) with the same whole recombinant MOG used for immunization (0.5 μg/ml). After blocking with 1% BSA, wells were incubated in duplicate with sera diluted between 1:1,000 and 1:100,000 in 1% BSA in PBS for 2 hr at room temperature. After washing with PBS, incubation with HRP‐conjugated goat anti‐rat IgG polyclonal antibodies (1:5000; GE Healthcare, Chicago, IL, Cat# NA931, RRID: AB_772210) was performed for 2 hr at RT, followed by reaction with tetramethylbenzidine (TMB) substrate (eBioscience, San Diego, CA) for 15 min at RT. The reaction was then arrested by the addition of 0.16 M H_2_SO_4_, and absorbance was measured at 450 nm. Pseudo‐quantification of sera anti‐MOG titers was achieved by comparison of all samples to a commercial anti‐MOG antibody (AnaSpec Inc., San Jose, CA; EGT Group Cat# 55914, RRID: AB_10730308), and use of sera dilutions falling within the linear range of the ELISA assay.

### 2.9 Cell‐based anti‐MOG antibody assay

Anti‐MOG antibodies were determined using a cell‐based assay with MOG transfected HEK293 cells. Briefly, HEK293 cells were transfected with either pcDNA™6.2/C‐EmGFP‐GW/TOPO™ expression plasmid (Invitrogen, Karlsruhe, Germany) containing full‐length human MOG or pcDNA™6.2/C‐EmGFP‐GW/TOPO™ only (empty vector [EV], control). Surface expression of MOG was verified by flow cytometry and confocal microscopy with the anti‐MOG monoclonal antibody 8‐18C5 in combination with the secondary APC‐labeled goat anti‐mouse IgG antibody (BioLegend). For the detection of MOG‐specific antibodies in rat sera, cells were incubated for 15 min on ice with rat sera diluted 1:400 in the growth medium. Cells were subsequently washed three times with wash buffer (PBS plus 2% FBS) and APC‐labeled goat anti‐rat IgG antibody (1:200, Invitrogen) was added for 15 min on ice, followed by two additional washing steps. Median fluorescence intensity (MFI) was analyzed on a FACS Canto (BD Bioscience; San Jose, CA) and the MFI ratio of HEK^MOG^ versus HEK^EV^ was calculated and plotted using GraphPad Prism® software.

### 2.10 Knockdown of αB‐crystallin

Predesigned 29‐mer shRNA‐containing retroviral vectors were purchased from OriGene (Rockville, MD) containing either the αB‐crystallin‐specific sequence (5′–3′) AGATGCGTATGGAGAAGGACAGGTTCTCT (Catalog Nr. TG709545) or a noneffective scrambled sequence (Catalog Nr. TR30013). Lentiviral particles were generated in HEK 293LTV cells using a Lenti‐vpak packaging kit (OriGene; Catalog Nr. TR30037). Oligodendrocyte cultures were infected 4 days after switching from proliferation to differentiation media with a 1:100 dilution of lentivirus in media and left for a further 12 days until experiments were performed.

### 2.11 RNA isolation, cDNA synthesis, and quantitative real‐time PCR

Total RNA was extracted from dissected optic nerve heads and distal optic nerves (segments of approximately 1 mm in length) using RNeasy Plus Micro Kit (Qiagen, Hilden, Germany) and then transcribed into cDNA using First Strand cDNA Synthesis Kit (Roche) with oligo(dT) primers. Quantitative Real‐Time PCR was subsequently performed using an ABI 7300 thermal cycler (Applied Systems, University Park, IL) with RT2 SYBR Green ROX mastermix (Qiagen). Relative quantification of gene expression was determined by comparison of threshold values, normalized to β‐actin, calculated by 2(−ΔΔC_t_). The following primers were used: αB‐crystallin—forward 5′‐GACCGGCTAACCGACTCTAC‐3′, reverse 5′‐GGTGCTCTCCGAAGAACTGG; β‐actin—forward 5′‐CTCTGTGTGGATTGGTGGCT‐3′, reverse 5′‐GGGTGTAAAACGCAGCTCAG‐3′.

### 2.12 Statistical analyses

All data are presented ± SEM. Statistical comparisons were made using SigmaPlot 13 (Systat Software GmbH, San Jose, CA). For comparing two experimental groups, data were assessed for normality using the Shapiro–Wilk Test, followed by either a two‐tailed Student's *t* test or by Mann–Whitney rank sum test. Multiple experimental groups were analyzed using one‐way analysis of variance (ANOVA) with post hoc Dunnett's test. A *p* value of <.05 was considered to be statistically significant.

## 3 RESULTS

### 3.1 αB‐Crystallin expression is up‐regulated during the preclinical phase of autoimmune optic neuritis

**Figure 1:**
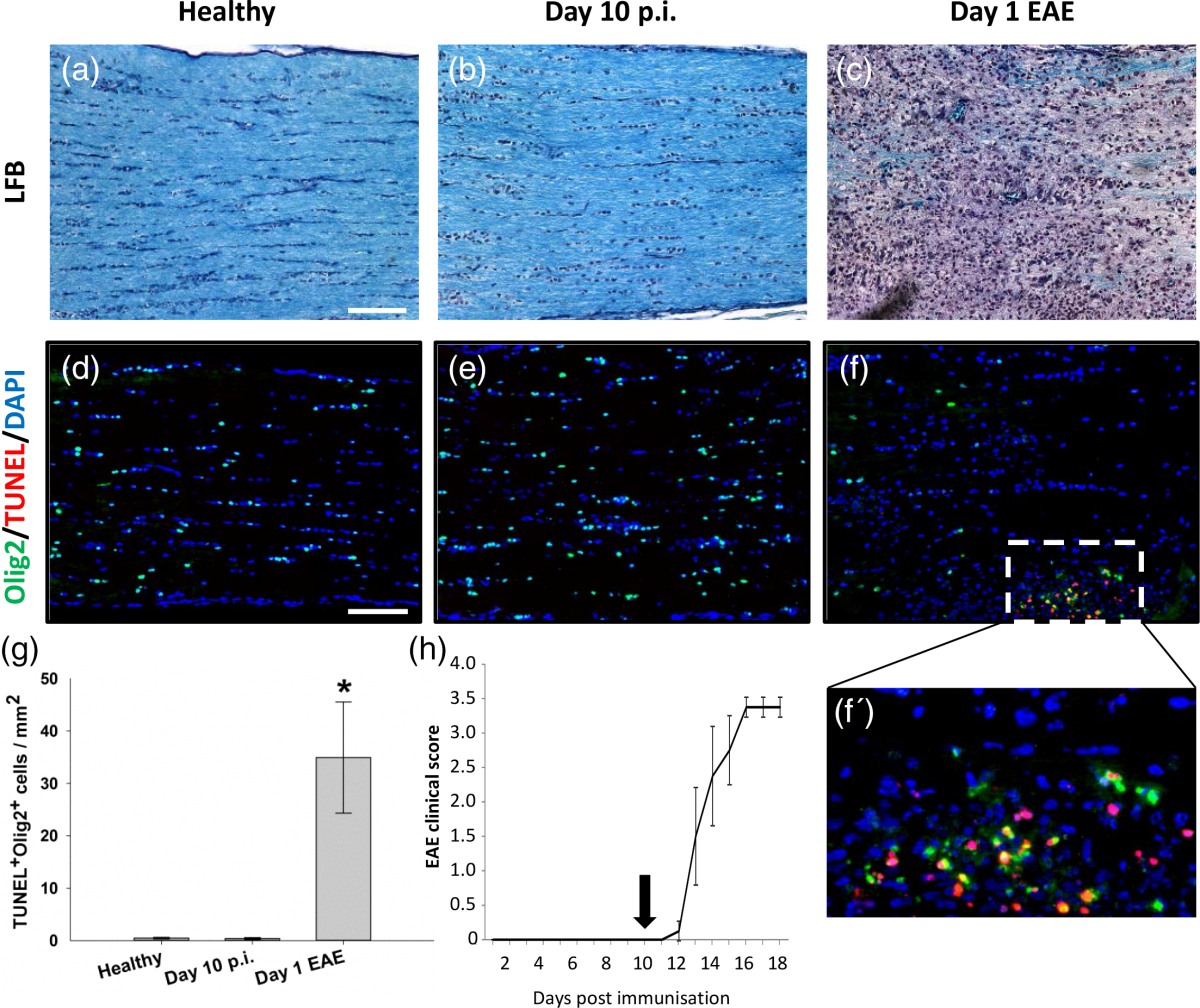
Optic nerve oligodendrocyte apoptosis begins at onset of clinical EAE. Representative images of optic nerves following LFB‐staining of myelin (blue) from (a) healthy, (b) 10 days post immunization (p.i.) and (c) animal on first day of EAE onset. Representative immunohistochemical images of TUNEL‐labeled optic nerves from (d) healthy, (e) day 10 p.i., and (f) day 1 of EAE (TUNEL, red; counterstained with Olig2 [green] to identify oligodendrocytes). Apoptotic oligodendrocytes could only be observed within the inflamed optic nerve at day 1 of EAE (f, and enlarged region in f'). (g) Quantification of TUNEL‐positive Olig‐2‐positive cells throughout the optic nerves (*n* = 6 optic nerves per time‐point). (h) A typical EAE time‐course of MOG‐immunized animals, with day 10 p.i. time‐point indicated (*n* = 4). Scale bars in (a) 50 μm and (d) 100 μm. *p < .01 compared to healthy, ANOVA

AON is characterized by immune cell infiltration and demyelination of the optic nerves which typically occurs, in BN rats immunized with MOG, from around 12 days following disease induction, and correlates with the onset of spinal cord symptoms associated with EAE. However, it is known that degenerative and pathological changes within the optic system begin prior to the onset of AON/EAE (Fairless et al., 2012). To confirm that lesion formation and loss of oligodendrocytes within the optic nerve conformed to this time‐course LFB staining was performed to visualize the myelin. No loss of myelin was observed within the preclinical phase (day 10 post immunization; Figure 1b), but wide‐spread demyelination was apparent on the first day that clinical EAE symptoms were observed (day 13.64 ± 0.68; Figure 1c), correlating with immune cell infiltration indicated by the hyper‐cellularity revealed by hematoxylin nuclear staining. Next, apoptosis of oligodendrocytes was investigated by co‐staining optic nerves sections with an antibody against the oligodendrocyte marker Olig2 in combination with TUNEL staining. No TUNEL positive cells were observed during the preclinical disease stages but were apparent at day 1 of EAE (Figure 1f,g; 34.93 ± 10.62, *p* = .002, ANOVA). In general, there were fewer Olig2‐positive cells distributed along day 1 EAE optic nerves, presumably resulting from a loss of oligodendrocytes at disease onset. However, many of those that were detectable were clustered in regions of TUNEL‐positivity (Figure 1f′) indicating areas of active tissue damage.

**Figure 2:**
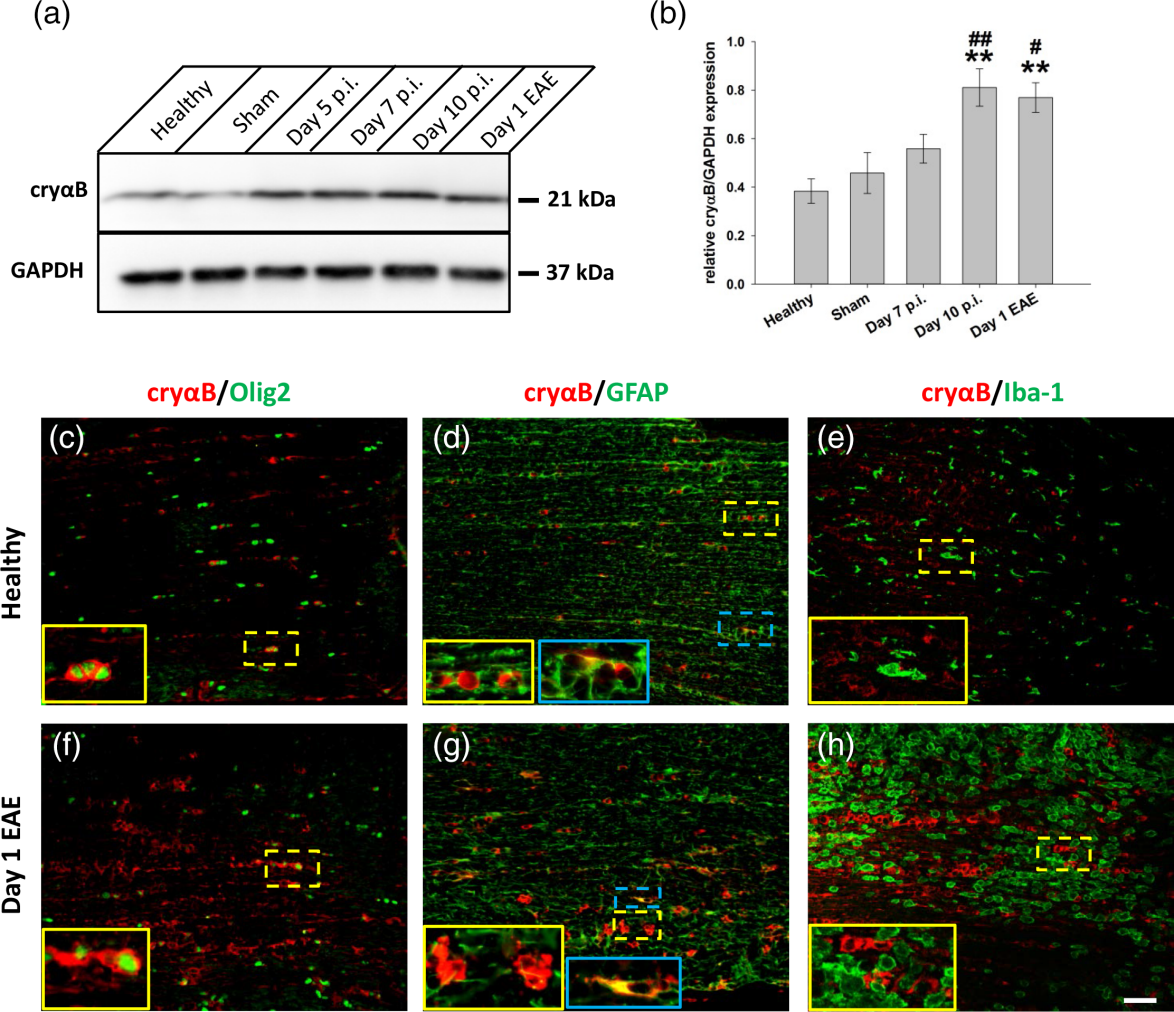
αB‐crystallin expression increases throughout course of AON. (a) Western blot of optic nerve lysates from different time points throughout time‐course of AON using antibodies against αB‐crystallin (cryαB) and GAPDH. (b) Quantification revealed a two‐fold increase in cryαB starting already during the late induction phase of AON (day 10 p.i.; *n* = 4 pooled optic nerves per time point). (c–h) Immunostaining against cryαB with counterstaining for different glial cell markers, performed on optic nerve sections from healthy (c–e) and animals from day 1 of EAE (f–h). CryαB‐positive staining was mostly associated with oligodendrocytes (Olig2^+^ cells; c and f), whereas only modest co‐localization could be observed with GFAP^+^ astrocytes (d and g, blue inserts). No co‐localization could be observed with Iba‐1, a marker for microglia/macrophages (e and h). **p < .01 compared to healthy; and ^#^ *p* < .05, ##*p* < .01 compared to sham, ANOVA). Scale bar = 100 μm

Next, the expression of the heat‐shock protein, αB‐crystallin was investigated. αB‐Crystallin has been reported to be elevated in models of ischemia or neuroinflammation, such as stroke and EAE (Arac et al., 2011; Ousman et al., 2007), and also in MS (Bajramovic et al., 1997; Van Noort et al., 2010). Since its accumulation in MS lesions was reported in preactive lesions which have been proposed to represent a stage in early lesion formation, we wished to determine the time‐course of αB‐crystallin expression during AON. Western blot analysis of optic nerve lysates revealed that αB‐crystallin expression increases as the disease progresses, becoming significantly elevated by day 10 post immunization (Figure 2b**,** healthy = 0.38 **±** 0.05, day 10 = 0.81 **±** 0.08 relative to GAPDH, *p* = .002 compared to healthy, ANOVA) during the preclinical disease phase. To determine in which cell type αB‐crystallin was expressed, co‐staining of optic nerve tissue sections was performed with antibodies against αB‐crystallin in combination with markers against oligodendrocytes (Olig2), astrocytes (GFAP), and microglia/macrophages (Iba1). Expression was primarily seen in oligodendrocytes and also in some astrocytes, but not in microglia/macrophages (Figures 2c–e). In later disease stages, these same cell types were still responsible for expression, although αB‐crystallin expression was more pronounced.

### 3.2 αB‐crystallin up‐regulation occurs predominantly within the optic nerve head

**Figure 3:**
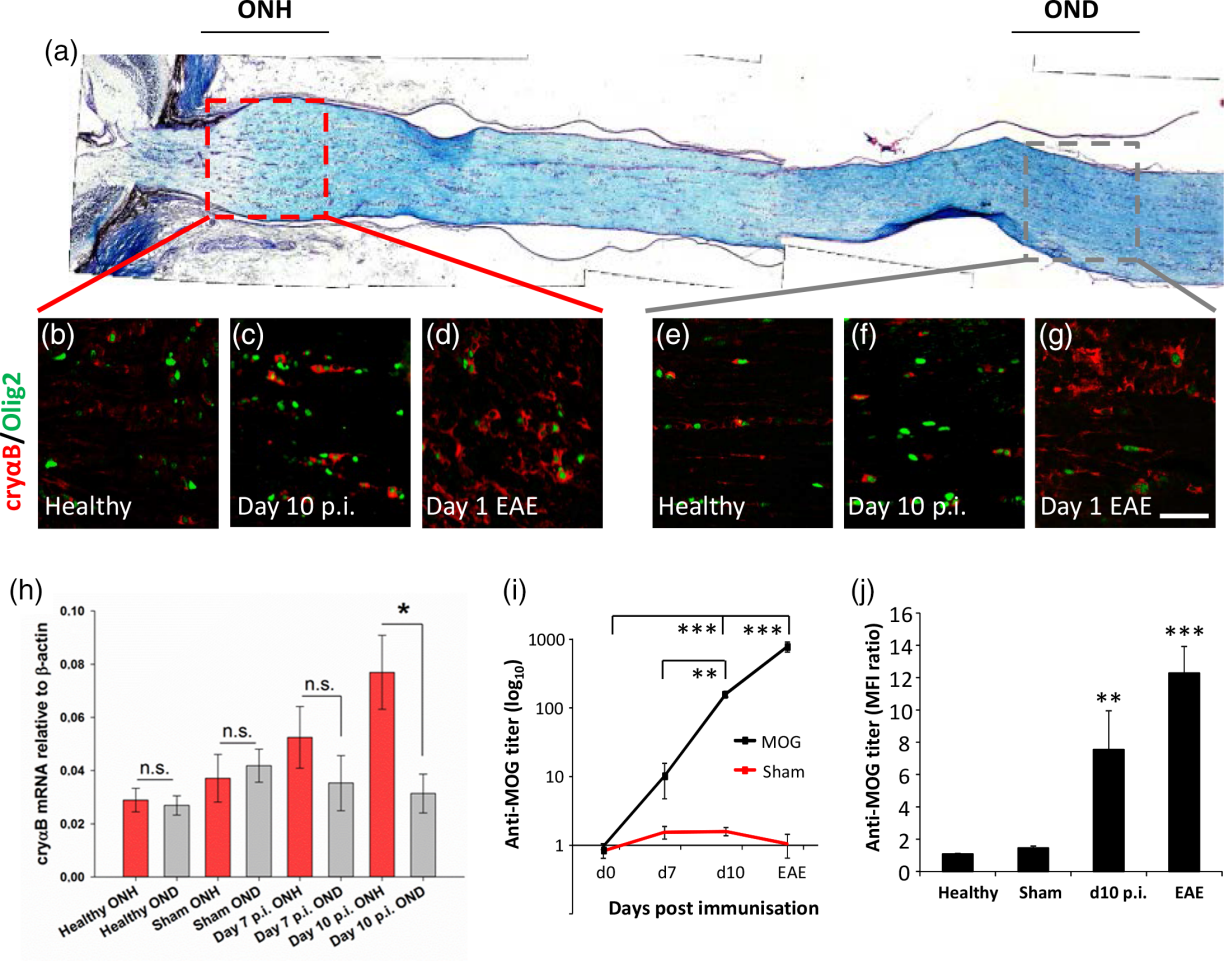
αB‐crystallin expression increases predominately in the optic nerve head during AON. (a) Longitudinal histochemical staining (LFB) of a healthy optic nerve, indicating regions of the optic nerve head (ONH) and distal optic nerve (OND) assessed in further detail. Example images of (b–d) the optic nerve head and (e–g) the distal optic nerve from (b, e) healthy, (c, f) day 10 p.i., and (d, g) animals at the onset of EAE. All were immunostained with antibodies against αB‐crystallin (cryαB) and Olig2. (h) Changes in optic nerve gene expression of cryαB were addressed by qPCR, comparing the optic nerve head (ONH) with the distal optic nerve (OND) during AON. There was a significant increase of cryαB mRNA in the optic nerve head compared to distal segments of optic nerve at day 10 p.i. (*n* = 11 for healthy and d10 pi optic nerves, and 8 for sham and d7 pi optic nerves); (i) anti‐MOG antibody titers in MOG (black line) and sham (red line) immunized animals at different time points (*n* = 4 per time‐point) were analyzed by ELISA. (j) The binding capacity of anti‐MOG antibodies to native huMOG was assessed with 1:400 sera dilutions by flow cytometry using HEK293 cells transfected with either full length MOG or empty vector, and is depicted as the median fluorescence intensity (MFI) ratio of HEK^MOG^ versus HEK^EV^ (*n* = 10 per time‐point). *p < .05, **p < .01, ***p < .001, ANOVA. Scale bar = 100 μm

To determine whether increased oligodendrocyte αB‐crystallin expression was uniformly distributed or whether it was restricted to specific anatomical regions of the optic nerve, optic nerves were carefully dissected together with the eye to obtain longitudinal sections containing a complete optic nerve head (Figure 3a). Sections were then stained with antibodies against αB‐crystallin and Olig2. Focus was made on the initial myelinated region of the optic nerve head and was compared with more distal regions of the optic nerve. Although both regions had elevated tissue staining at day 1 of EAE (Figure 3d,g), at day 10 p.i., only the optic nerve heads had elevated staining (Figure 3c). This was confirmed by qPCR analysis of αB‐crystallin mRNA levels isolated from approximately 1 mm thick optic nerve tissue segments to enrich for the optic nerve heads in comparison with similar sized optic nerve pieces dissected from a more distal position (Figure 3h; at day 10, the optic nerve head had αB‐crystallin relative to β‐actin of 0.08 ± 0.01 compared to the distal optic nerve with a value of 0.03 ± 0.007, *p* = .03, Mann–Whitney).

**Figure 4:**
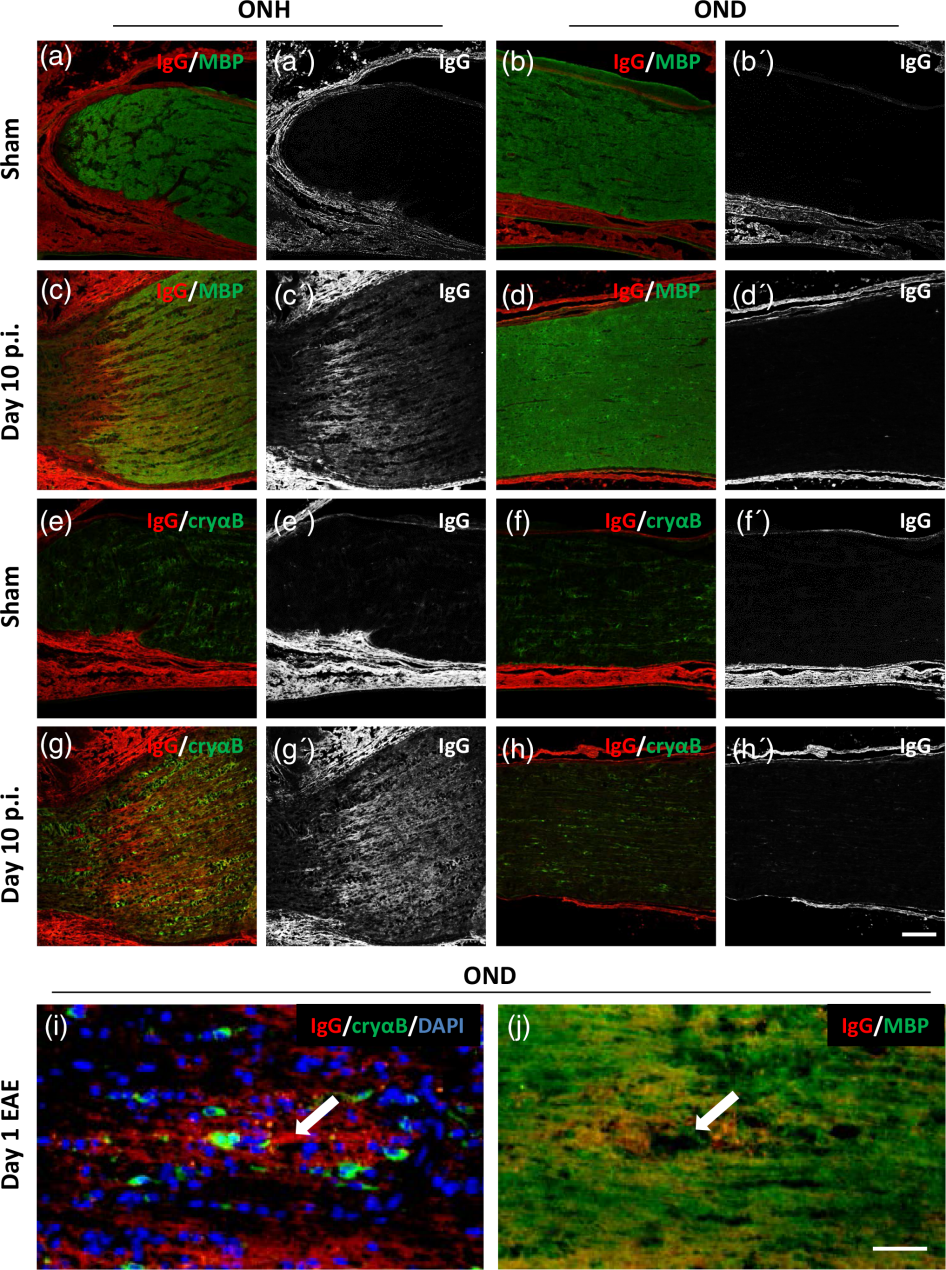
αB‐crystallin expression correlates with areas of anti‐MOG antibody deposition. Immunohistochemistry of optic nerve sections from animals 10 days following immunization with either (a, b, e, f) sham or (c, d, g, h) MOG. Optic nerve head (ONH; a, c, e, g) and distal optic nerve (OND; b, d, f, h) were stained with antibodies against (a–d) IgG deposition and myelin basic protein (MBP); and (e–h) IgG deposition and αB‐crystallin (cryαB). Single channel images of IgG deposition are shown in a'–h'. (i, j) Serial optic nerve sections from a distal segment of optic nerve taken from an animal at day 1 of EAE, stained against (i) IgG deposition, cryαB, and DAPI; and (j) IgG deposition and MBP. White arrow in i and j indicates location of an inflamed blood vessel. Scale bars (h') = 100 μm, (j) = 50 μm

Since we have previously reported that the optic nerve head during AON is more susceptible to deposition of (autoimmune) antibody (Fairless et al., 2012), we next wished to determine if there was a correlation between IgG deposition and αB‐crystallin expression. Anti‐MOG antibody levels were assessed in sera samples taken during the progression of AON. These increased as the disease progressed in MOG‐immunized rats, but not in those receiving sham‐immunization, becoming significantly elevated at day 10 p.i. (Figure 3i; 0.96 +/− 0.09 at d0 p.i. vs. 159.45 +/− 15.27 at d10 p.i., *p* < .001, ANOVA). Similarly, significantly elevated anti‐MOG titers were detected at day 10 p.i. (Figure 3j; MFI ratio 7.54 ± 2.41, compared to 1.08 ± 0.04 at d0 p.i.; *p* = .0098, ANOVA) using flow cytometry to assess the binding capacity of anti‐MOG antibodies to huMOG‐expressing HEK cells, demonstrating their ability to bind the fully folded native confirmation of human MOG. Thus, increases in anti‐MOG antibody levels correlate with the time‐point that increases in αB‐crystallin expression became significant (Figures 2b and 3h). Immunohistochemical staining of optic nerve head and distal optic nerve tissue sections demonstrated αB‐crystallin elevation at day 10 post immunization in the optic nerve heads of MOG‐immunized rats (Figure 4g) but not sham‐immunized rats (Figure 4e). This correlated with the same regions displaying observable IgG deposition (Figure 4c',g'). Similarly, in distal optic nerves where no IgG deposition was seen, αB‐crystallin expression was not easily visible. However, in later disease sections (Figure 4i, j; day 1 EAE) when the blood–brain barrier of the optic nerve breaks down permitting entry of infiltrating immune cells (note high density of DAPI‐labeled nuclei in Figure 4i), areas of IgG deposition could be seen in the vicinity of what is probably a disrupted blood vessel permitting antibody entry. A decrease in myelin staining could also be seen in this area.

### 3.3 Passive transfer of sera results in αB‐crystallin upregulation

**Figure 5:**
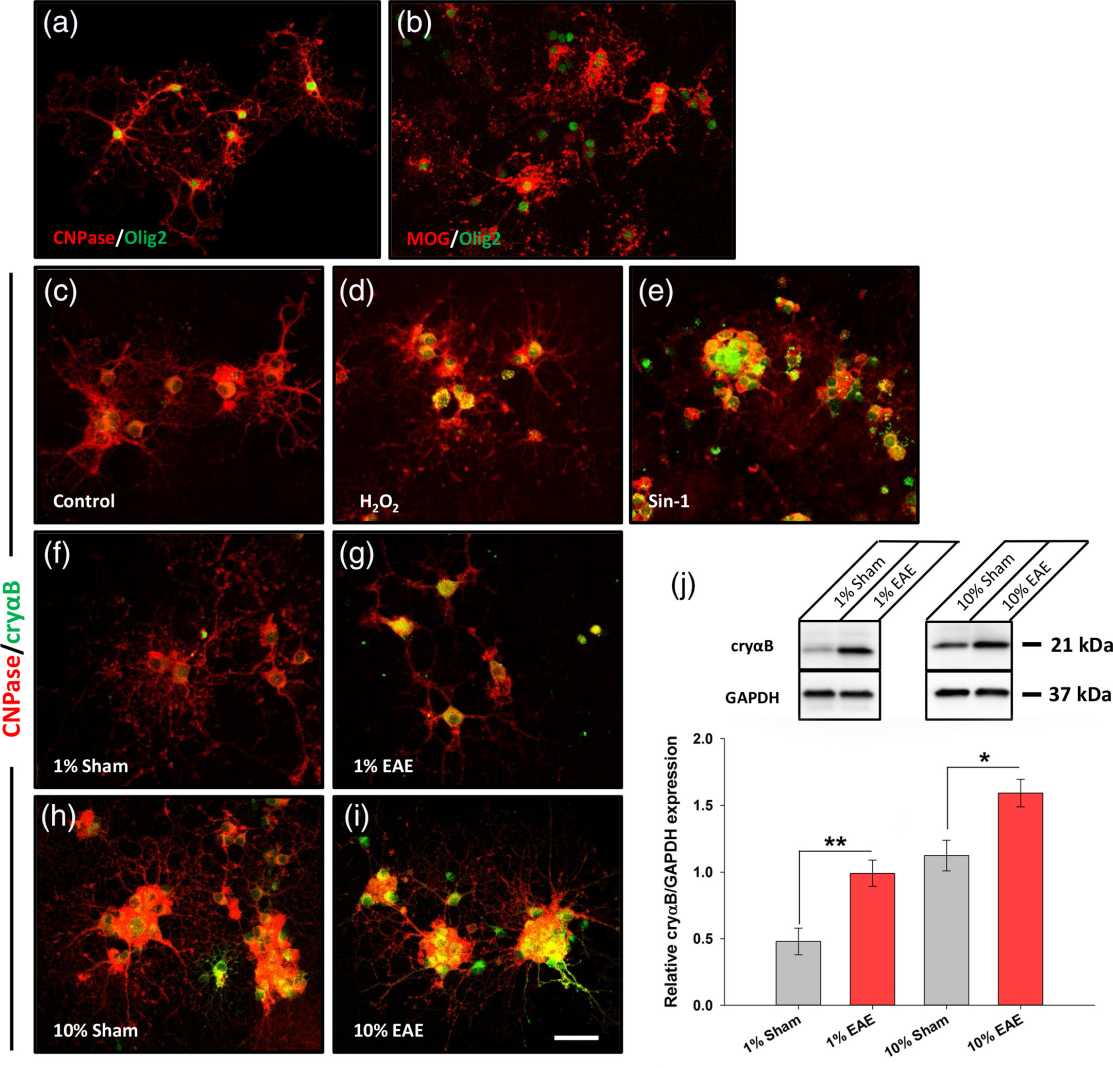
Treatment with EAE sera from MOG immunized rats leads to increased expression of αB‐crystallin in primary oligodendrocyte cultures. Immunocytochemistry showing mature oligodendrocytes expressing (a) CNPase and (b) MOG (red), co‐stained with Olig2 (green). Immunocytochemistry of oligodendrocytes with CNPase (red) and αB‐crystallin (cryαB; green) following (c) no treatment, or 90 min in (d) 100 μM H_2_O_2_ and (e) 100 μM Sin‐1 as positive controls for stress. Treatment with sera from (f, h) sham and (g, i) MOG immunized rats led to increased expression of cryαB (green). (j) Western blot and quantification revealed significant elevation of cryαB in MOG sera‐treated cultures than sham (*n* = 5 treated cultures for 1% serum treatment and *n* = 3 treated cultures for 10% serum treatment). Scale bar = 50 μm. *p < .05, Student's *t*‐test

Next, to determine if blood‐borne factors, such as circulating autoantibodies, were able to induce an up‐regulation in αB‐crystallin expression, oligodendrocyte progenitors were isolated from cortices and cultured in vitro. Afterward, the progenitors were differentiated into mature oligodendrocytes, as observed by their extension of numerous processes and confirmed by immunostaining for the myelin components CNPase and MOG (Figure 5a,b). Mature oligodendrocytes were then either left untreated (control) or treated with H_2_O_2_ or linsidomine (Sin‐1, a NO donor) for 90 min followed by overnight recovery to induce oxidative stress. This resulted in pronounced up‐regulation of αB‐crystallin expression as assessed by immunocytochemistry. Next, mature oligodendrocytes were treated with sera obtained from either sham‐immunized (d14 p.i.) or MOG‐immunized (d1 EAE) animals at the indicated dilution in culture media for 90 min. Although some up‐regulation of αB‐crystallin was detectable following treatment with sham sera, this was much higher following treatment with EAE sera (Figure 5f–i) and was confirmed by Western blot (Figure 5j; 1% sham (0.48 ± 0.1) vs. 1% EAE sera (0.99 ± 0.1), *p* = .007; 10% sham (1.12 ± 0.11) vs. 10% EAE sera (1.59 ± 0.1), *p* = .039; paired *t* tests).

**Figure 6:**
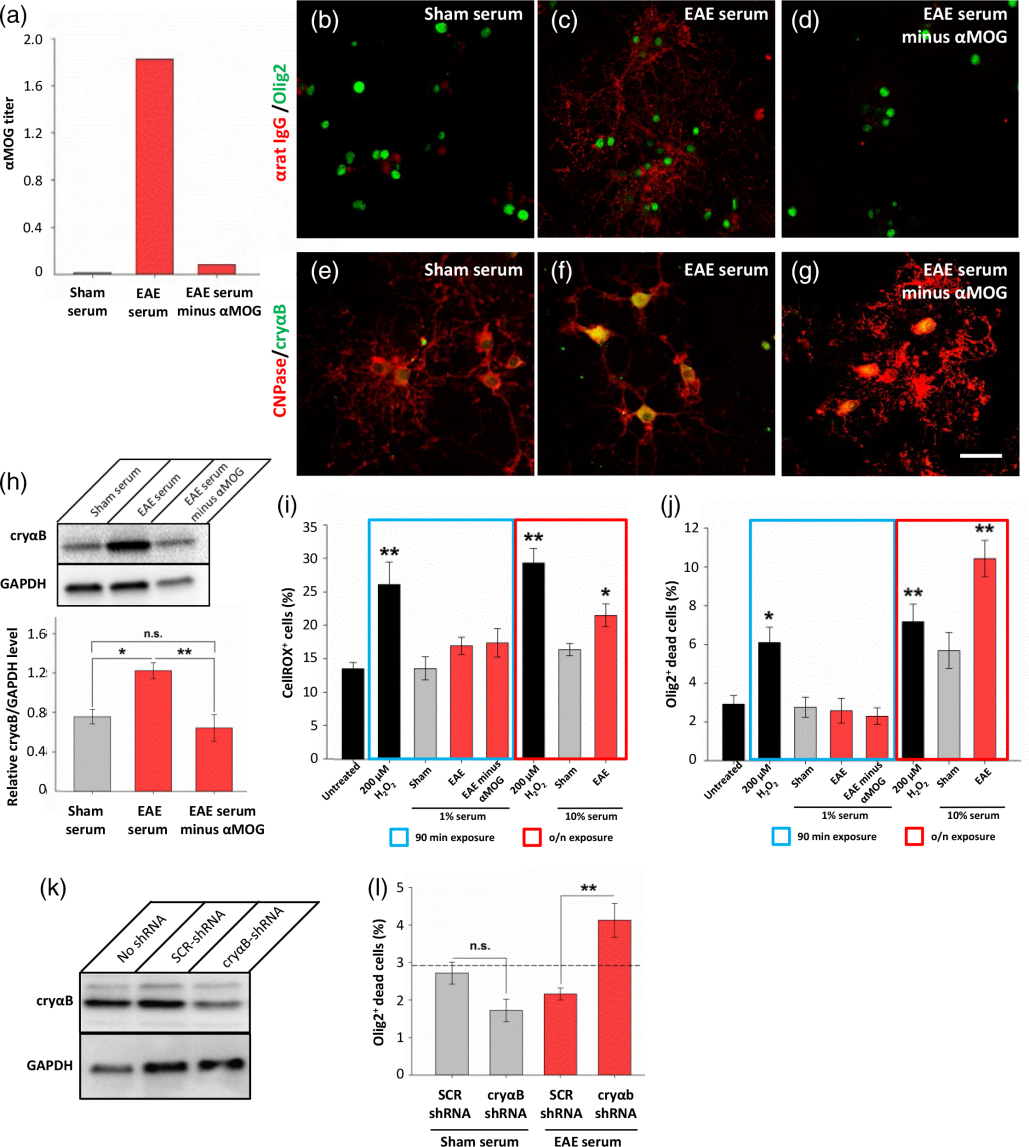
Anti‐MOG antibodies are necessary for EAE sera to induce αB‐crystallin upregulation in primary oligodendrocyte cultures. (a) ELISA of anti‐MOG antibody titers demonstrates efficacy of anti‐MOG antibody depletion. (b–d) EAE serum, but not that depleted of anti‐MOG antibodies, is able to immunolabel unfixed oligodendrocytes (red, anti‐IgG; green, olig2). (e–g) Oligodendrocyte cultures exposed to 1% sera for 90 min followed by overnight recovery were immunolabeled with antibodies against CNPase (red) and αB‐crystallin (cryαB, green). (h) Western blot with quantification showing upregulation of αB‐crystallin following 90 min exposure to 1% EAE serum, but not with EAE serum depleted of anti‐MOG antibodies. (i) Reactive oxygen species were quantified in oligodendrocyte cultures using CellROX assay. Cells exposed to H_2_O_2_ had increased ROX positivity, but not cells exposed to 1% serum. Overnight exposure of cells to 10% serum or H_2_O_2_ served as positive controls. (j) Oligodendrocyte toxicity was assessed using a fixable LIVE/DEAD assay demonstrating increased cell death upon exposure to H_2_O_2_, and also EAE serum when exposed overnight. (k) Lentiviral delivery of shRNA against αB‐crystallin successfully reduced αB‐crystallin expression in oligodendrocyte cultures compared to delivery of a scrambled control (SCR) shRNA sequence. All cultures were exposed to 10% EAE serum for 90 min followed by overnight recovery. (l) Oligodendrocytes treated with shRNA targeting αB‐crystallin had increased cell death upon 90 min exposure to 1% EAE sera compared to those treated with scrambled shRNA, or exposure to sham sera. Dashed line indicates the untreated control levels (as shown in panel j). Scale bar = 50 μm; *p < .05, **p < .01, ANOVA

To determine the influence of anti‐MOG antibodies on αB‐crystallin expression, we next depleted EAE serum of anti‐MOG antibodies using MOG‐conjugated Sepharose resin. Confirmation of antibody depletion was assessed by MOG‐ELISA (Figure 6a) as well as staining of fully differentiated oligodendrocyte cultures with sera (Figure 6b–d). Only sera containing anti‐MOG antibodies were able to immunolabel oligodendrocytes, but not sham sera or EAE sera following depletion of the anti‐MOG antibody fraction. Oligodendrocytes were next exposed to the sera samples for 90 min followed by overnight recovery, to mimic the “sublytic” conditions of the preclinical environment (i.e., presence of antibody but no oligodendrocyte death/myelin loss; Figures 1b,e,g and 3i). Both immunolabeling and Western Blot demonstrated an upregulation of αB‐crystallin in oligodendrocytes which were exposed to EAE serum, but not following depletion of anti‐MOG antibodies (1% EAE serum, 1.22 ± 0.08; 1% EAE serum depleted of anti‐MOG antibodies, 0.64 ± 0.13; *p* = .012, ANOVA; Figure 6h). Analysis of reactive oxygen species, a key component of tissue injury in inflammatory demyelinating lesions (Lassmann, 2014), as well as cell survival, was then performed. Reactive oxygen species levels were elevated in controls (exposure to H_2_O_2_ or overnight incubation in EAE serum) but not under sublytic conditions (Figure 6i). Similarly, cell death was not induced in oligodendrocytes exposed to sublytic sera conditions (Figure 6j).

In order to further our understanding regarding the function of αB‐crystallin upregulation, we next used shRNA to knock‐down its expression. Treatment of oligodendrocyte cultures with lentiviral particles carrying shRNA constructs previously demonstrated to reduce αB‐crystallin expression (Gangalum, Bhat, Kohan, & Bhat, 2016) similarly reduced expression of αB‐crystallin in cultures which were treated with EAE sera (Figure 6k). Cell survival of lentiviral‐transfected oligodendrocyte cultures was then assayed following exposure to sera. As before, control cultures were invulnerable to sublytic sera exposure, however a significant elevation in cell death was observed in oligodendrocytes which received αB‐crystallin shRNA and subsequently exposed to EAE serum (control cell death, 2.92 ± 0.44%; SCR shRNA 2.16 ± 0.16%, cryab shRNA 4.13 ± 0.45%, *p* = .005; ANOVA; Figure 6l). Thus knock‐down of αB‐crystallin sensitized the oligodendrocytes to EAE serum‐mediated toxicity.

**Figure 7:**
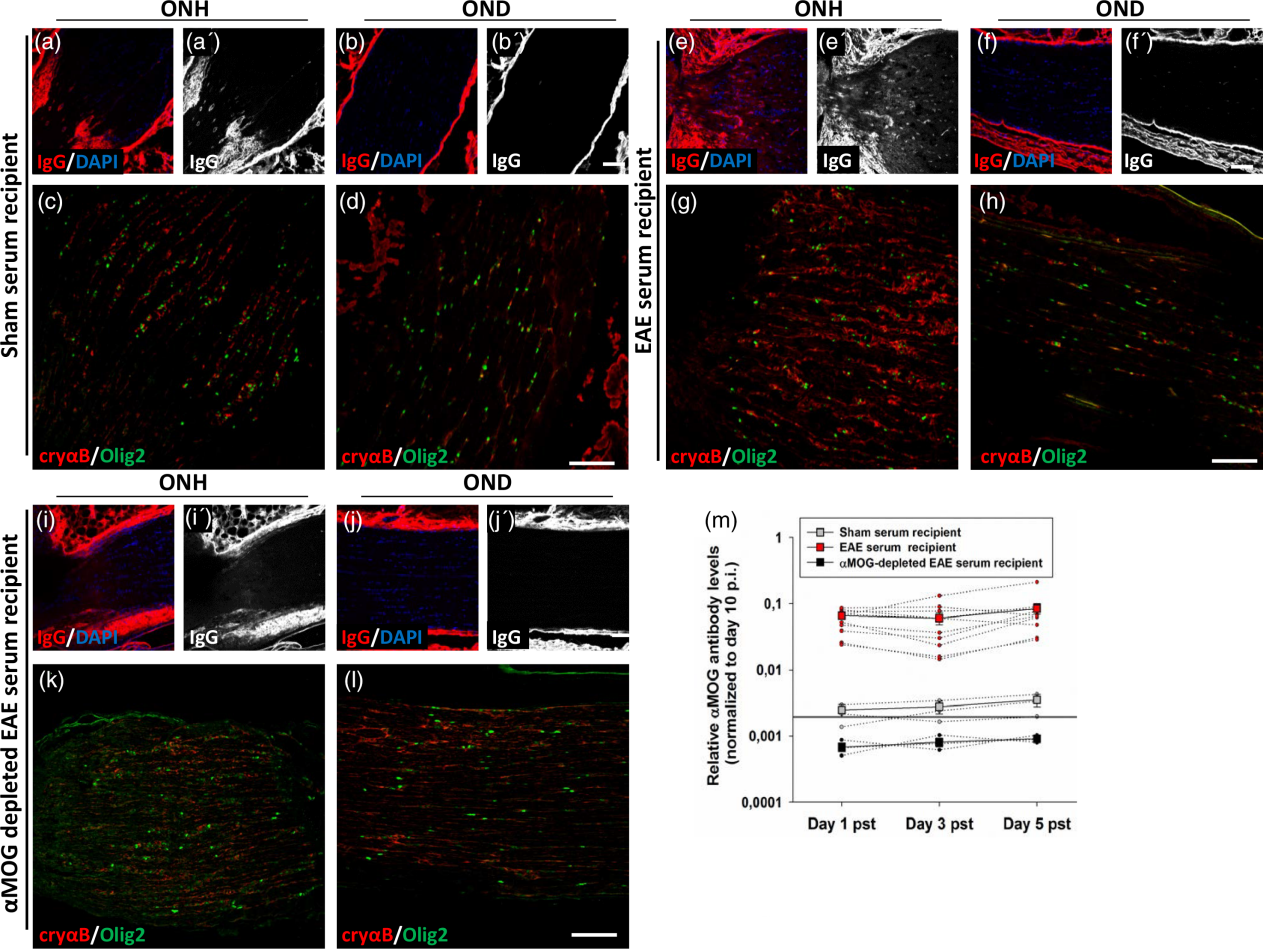
Passive transfer of EAE sera results in elevated αB‐crystallin expression within the optic nerve head of unimmunized, recipient rats. Immunohistochemistry of optic nerve head (ONH; a, c, e, g, i, k) and distal optic nerve (OND; b, d, f, h, j, l) regions from representative unimmunized rats receiving passive transfer by intravenous injection of sham (a–d) or MOG EAE (e–h) sera, or MOG EAE sera that had been depleted of anti‐MOG antibodies (i–l). Immunohistochemistry was performed against deposited IgG with (a, b, e, f, i, j) and without (a′, b′, e′, f′, i′, j′) DAPI counter‐labeling of nuclei, or against αB‐crystallin (cryαB, red) and Olig2 (green) (c, d, g, h, k, l). (m) Anti‐MOG ELISA was performed on sera samples obtained from recipient rats. Black line indicates the level of αMOG antibody in sham immunised donor rats (day 14 p.i.). Scale bars = 100 μm

Finally, we wished to determine whether passive transfer of sera from immunized rats with EAE would be sufficient to induce expression of αB‐crystallin in naïve, unimmunized recipient rats. To achieve this, sera was collected as before from either sham‐immunized (d14 p.i.) or MOG‐immunized rats (d1 EAE) and injected intravenously. Anti‐MOG antibody titers were then measured in sera samples taken from recipient animals as an indicator of successful transfer. Rats receiving sham sera had negligible anti‐MOG titers, as assessed by ELISA, whereas this was robustly elevated in rats receiving EAE sera (Figure 7m). Five days following passive transfer (day 5 pst), recipient rats were perfused and optic nerves were analyzed for deposition of IgG antibody and αB‐crystallin expression. Antibody deposition was only seen in the optic nerve heads of rats receiving EAE sera (Figure 7e′), but not in the distal optic nerve nor in optic nerves of rats receiving sham sera. Similarly, αB‐crystallin up‐regulation was only apparent within the optic nerve heads of rats receiving EAE sera, where it correlated with the regions of IgG deposition. To further clarify the impact of anti‐MOG antibodies on αB‐crystallin expression in vivo, we repeated passive transfer experiments using EAE sera that had been depleted of anti‐MOG antibodies. Similar to that seen in rats receiving sham sera, optic nerve heads had neither evidence of IgG deposition nor changes in αB‐crystallin expression (Figure 7i,k), demonstrating the requirement for anti‐MOG antibodies to induce upregulation of αB‐crystallin.

## 4 DISCUSSION

Here we report that an early stress response, as revealed by αB‐crystallin expression, could be detected in oligodendrocytes (and also astrocytes) during preclinical AON, where it was mostly restricted to the vicinity of the optic nerve head. It has previously been reported that the blood–brain barrier is incomplete in this region (Hofman, Hoyng, vanderWerf, Vrensen, & Schlingemann, 2001; Tso, Shih, & McLean, 1975), and we previously reported that blood‐borne proteins such as albumin, and also IgG, were able to gain access to the tissue parenchyma of the optic nerve head (Fairless et al., 2012). Similarly, we report here that passive transfer of sera from MOG‐immunized animals resulted in IgG deposition within this region, overlapping with the myelinated regions of axons, thus coming into contact with oligodendrocytes which displayed signs of cellular stress as indicated by αB‐crystallin up‐regulation. Similar observations were made in more distal regions of the optic nerve in the vicinity of disturbed blood vessels, indicating a similar cellular reaction to the entry of MOG EAE‐specific sera components, which in addition to cytokines and complement proteins, involve the function of anti‐MOG antibodies as indicated by our antibody depletion experiments. The role that anti‐MOG antibodies might be playing in this disease model is intriguing due to their association with other neurological conditions such as pediatric MS, neuromyelitis optica in AQP‐4 antibody‐negative cases and occasionally anti‐NMDA‐receptor encephalitis (Havla et al., 2017). Although the relationship of anti‐MOG antibodies to adult MS has not been so clear, recent studies have reported that they are strongly associated with bilateral optic neuritis in adults (Ramanathan et al., 2014). Interestingly, MOG‐antibody‐associated optic neuritis was shown to have a strong correlation with optic nerve head swelling (over 50% of reported patients) which was absent in MS‐associated optic neuritis (Ramanathan et al., 2016). This fits with the observations we previously reported in rat AON where increased numbers of activated microglia were particularly apparent in the vicinity of the optic nerve head (Fairless et al., 2012).

At present, it is unclear in our model what the stress response we report represents—however, due to evidence gleaned from other injury models, it may well be part of a protective process. αB‐crystallin is up‐regulated following various injuries and insults such as stroke and oxidative damage, as well as in models of neuroinflammation and age‐related macular degeneration (Chis et al., 2012; Fittipaldi et al., 2015; Goldbaum, Riedel, Stahnke, & Richter‐Landsberg, 2009; Ke et al., 2013; Shao et al., 2013; Zhou et al., 2014). It appears to form part of a protective reaction to injury since it can act to regulate apoptosis through interaction with proteins such as p53, Bax, BclX_S_, and caspase‐3 (Hu et al., 2012; Liu, Li, Tao, & Xiao, 2007; Mao, Liu, Xiang, & Li, 2004), and to modulate the immune response (Bsibsi et al., 2013; Ousman et al., 2007; Quach et al., 2013). This has been demonstrated in knock‐out mice where a lack of αB‐crystallin resulted in exacerbation of EAE, and similarly, systemic administration of soluble αB‐crystallin ameliorated disease (Ousman et al., 2007). This view is further supported by our observation that shRNA‐mediated knock‐down of αB‐crystallin rendered previously invulnerable oligodendrocyte cultures susceptible to toxicity induced by short exposure to EAE sera.

However, αB‐crystallin may also play a detrimental role in disease development. It was originally reported to potentially be an autoantigen in MS (van Noort et al., 1995), although subsequent investigation did not support a clear connection with the pathology of either MS or EAE (Van Noort, Verbeek, Meilof, Polman, & Amor, 2006; Wang et al., 2006). In addition, although αB‐crystallin has been reported to induce a protective microglial response (Bsibsi et al., 2013), in combination with IFNγ, possibly deriving from infiltrating T cells, microglia can be reprogrammed to form a robust pro‐inflammatory response, facilitating demyelination (Bsibsi et al., 2014). Thus, its function appears to depend on the immune environment in which it is expressed, and may reflect an initial protective response during lesion formation, but later having limited effectiveness or even acting as a driving force behind the immune response following infiltration of peripheral immune cells.

In conclusion, we report that αB‐crystallin expression reports an early stress response in oligodendrocytes during the preclinical stage of AON, which correlates with areas of IgG deposition. This additionally demonstrates that the optic nerve head may represent a region of particular vulnerability due to its incomplete isolation from the vasculature, and thus may represent an Achilles' heel in the otherwise immune privileged CNS.

## CONFLICT OF INTEREST

The authors declare no competing financial interests.
